# Causal role of immune cells in bipolar disorder: a Mendelian randomization study

**DOI:** 10.3389/fpsyt.2024.1411280

**Published:** 2024-08-16

**Authors:** Mengxuan Wang, Shuo Wang, Guoshan Yuan, Mingzhou Gao, Xiyan Zhao, Zhenhan Chu, Dongmei Gao

**Affiliations:** ^1^ Department of Traditional Chinese Medicine, Shandong University of Traditional Chinese Medicine, Jinan, China; ^2^ Department of Intelligent and Information Engineering, Shandong University of Traditional Chinese Medicine, Jinan, China; ^3^ Innovation Research Institute of Traditional Chinese Medicine, Shandong University of Traditional Chinese Medicine, Jinan, China; ^4^ Department of Foreign Studies, China University of Petroleum (East China), Qingdao, China

**Keywords:** bipolar disorder, immune cells, Mendelian randomization study, causal association, SNP

## Abstract

**Background:**

The understanding of the immunological mechanisms underlying bipolar disorder (BD) has enhanced in recent years due to the extensive use of high-density genetic markers for genotyping and advancements in genome-wide association studies (GWAS). However, studies on the relationship between immune cells and the risk of BD remain limited, necessitating further investigation.

**Methods:**

Bidirectional two-sample Mendelian Randomization (MR) analysis was employed to investigate the causal association between immune cell morphologies and bipolar disorder. Immune cell traits were collected from a research cohort in Sardinia, whereas the GWAS summary statistics for BD were obtained from the Psychiatric Genomics Consortium. Sensitivity analyses were conducted, and the combination of MR-Egger and MR-Presso was used to assess horizontal pleiotropy. Cochran’s Q test was employed to evaluate heterogeneity, and the results were adjusted for false discovery rate (FDR).

**Results:**

The study identified six immune cell phenotypes significantly associated with BD incidence (*P*< 0.01). These phenotypes include IgD- CD27- %lymphocyte, CD33br HLA DR+ CD14- AC, CD8 on CD28+ CD45RA+ CD8br, CD33br HLA DR+ AC, CD14 on CD14+ CD16+ monocyte, and HVEM on CD45RA- CD4+. After adjusting the FDR to 0.2, two immune cell phenotypes remained statistically significant: IgD-CD27-% lymphocyte (OR=1.099, 95% CI: 1.051-1.149, *P* = 3.51E-05, FDR=0.026) and CD33br HLA DR+ CD14-AC (OR=0.981, 95% CI: 0.971-0.991, *P* = 2.17E-04, FDR=0.079). In the reverse MR analysis, BD significantly impacted the phenotypes of four monocytes (*P*< 0.01), including CD64 on CD14+ CD16+ monocyte, CD64 on monocyte, CX3CR1 on CD14- CD16-, CD64 on CD14+ CD16- monocyte. However, after applying the FDR correction (FDR < 0.2), no statistically significant results were observed.

**Conclusions:**

This MR investigation reveals associations between immune cell phenotypes, bipolar disorder, and genetics, providing novel perspectives on prospective therapeutic targets for bipolar disorder.

## 1 Introduction

Bipolar disorder, a lifelong and recurrent disorder, is characterized by alternating episodes of mania or hypomania and depression (1). Symptoms typically begin between ages 15 and 25 (2), and in severe instances, it can result in cognitive impairment (3), disability (4), and potentially suicide (5). It has been reported that individuals with BD have a suicide risk ranging from 4% to 19%, with 20% to 60% attempting suicide at least once in their lives (6). The etiology of BD remains uncertain, encompassing genetic predispositions, neurotransmitter activity, environmental influences, and immunological mechanisms (7, 8). Currently, lithium is the preferred treatment for BD (9), with cognitive therapy and electroconvulsive therapy as alternatives (10, 11). Timely identification and intervention assist individuals with BD in managing symptoms before severe problems arise, hence improving the prognosis (12). Recently, there has been a notable rise in BD incidence, resulting in an escalating worldwide impact (13).

The immune system and inflammation play significant roles in the pathology of various mental diseases (14). Recent research has demonstrated that the pathophysiology of BD is significantly influenced by inflammatory responses and immune modulation (11). There is a complex association between BD and immune inflammation mechanisms. Meta-analyses have shown increased levels of inflammatory markers, including C-reactive protein, tumor necrosis factor (TNF)-α, and interleukin (IL)-6, in individuals with BD (15). However, one study indicated that inflammatory markers such as interleukin-1 receptor antagonists and TNF-α may vary based on the emotional state of BD, such as normal mood, manic episodes, or depression (16). The longitudinal study has reported that elevated baseline levels of circulating inflammatory markers are associated with an increased risk of developing BD during follow-up (17). Additionally, a MR study has provided weak evidence suggesting that IL-13 and IL-17 may have a protective effect on BD patients (18).

Previous studies have demonstrated a significant association between BD and lymphocytes. Significant differences in T cell subgroups exist between BD patients and healthy controls (19). Additionally, the percentages of total T cells, CD4+ T cells, and CD71+ B cells in BD patients were significantly higher than those in the healthy control group (20). Nevertheless, it is important to acknowledge that the cause-and-effect connection between T cell imbalance and BD remains uncertain (21). Furthermore, a large-scale cross-sectional survey suggests that the ratios of neutrophils to lymphocytes and monocytes to lymphocytes are associated with an increased risk of developing BD (22). Additionally, existing evidence indicates a higher prevalence of autoimmune disorders, such as autoimmune thyroiditis, among individuals with BD, as well as an increased risk of developing BD among those with autoimmune disorders (23). Furthermore, laboratory research findings indicate that lithium treatment is associated with an increased production of natural killer cells induced by circulating cytokines (24). Randomized controlled trials have demonstrated the efficacy of boosting T cell system therapy in addressing immune-inflammatory abnormalities linked to BD and improving antidepressant responsiveness (25). A review article suggests utilizing inflammatory and immune response indicators to monitor the progression of bipolar disorder in patients (26). However, most existing evidence is based on observational studies, and research on the relationship between BD and immune cells may be limited by confounding variables and reverse causality.

As one of the most heritable psychiatric disorders, BD is expected to see a substantial increase in the number of relevant genetic loci in future large-scale studies. This is due to advancements in technology and methodologies, along with the adoption of international consortia and extensive population biobanks, enhancing genetic prediction for BD (27). Additionally, evidence of familial heritability has been discovered for BD (28), focusing on candidate genes such as BDNF, CLOCK, COMT, and DAOA to explore the clinical genetics of BD (7, 29). The review summarizes approaches aimed at identifying safer and more effective medications for individuals with BD through pharmacogenetics research (30). Hence, this study employs GWAS to investigate the complex interplay among individuals with BD, immune inflammation, and genetic predispositions, with the goal of identifying potential therapeutic targets to ameliorate BD symptoms.

MR is a method of causal inference that uses genetic variation as an instrumental variable. It utilizes the natural random allocation of genotypes to infer the effect of biological factors on disease phenotypes (31). MR methods have gained extensive application in causal inference within observational studies because genetic variations are inherent and less susceptible to common confounding factors such as environmental and social elements (32). Prior observational research has suggested an association between immune cell characteristics and BD. This study conducted a comprehensive two-sample MR analysis to investigate the causal relationship between immune cells and BD.

## 2 Materials and methods

### 2.1 Study design

**Figure 1:**
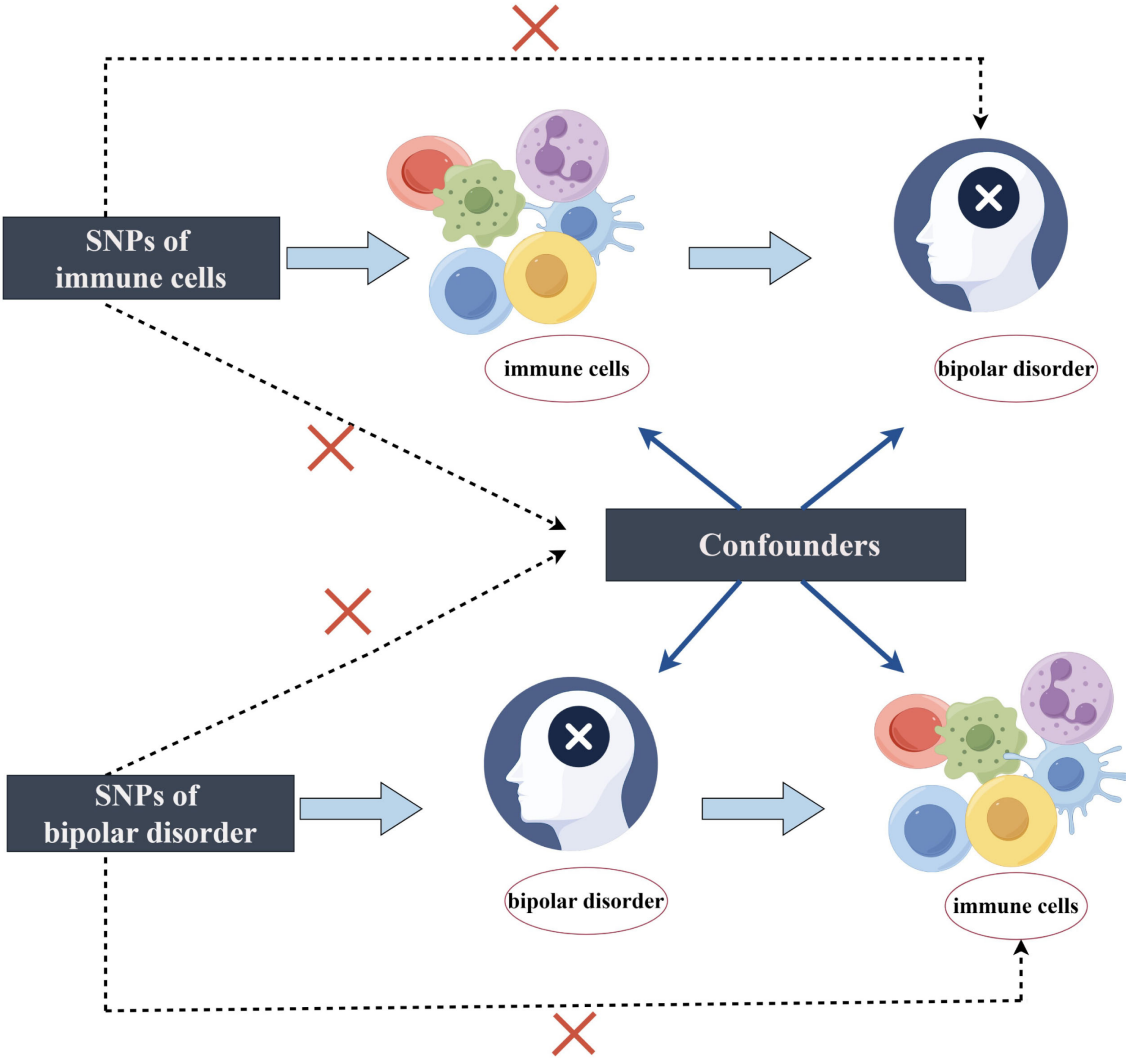
The design of bidirectional Mendelian randomization (MR) study by Figdraw.

A two-sample MR analysis was used to assess the causal association between 731 immune cell characteristics (7 sets) and BD. Genetic variations are utilized as instrumental variables (IVs) to represent risk factors in MR. This approach requires three essential assumptions to establish causal inference. (1) The IVs in this study are genetic variations significantly related to exposure. (2) IVs are independent of both known and unknown confounding influences. (3) IVs impact the outcome solely through exposure, not through alternative pathways. **Figure 1** illustrates the analysis workflow. The relevant institutional review board granted approval for this study.

### 2.2 Genome-wide association study data sources for BD

The GWAS summary statistics for BD were obtained from the Psychiatric Genomics Consortium (PGC) (33). The study conducted GWAS on 413,466 individuals of European descent (Ncase = 41,917, Ncontrol = 371,549). BD patients met the international consensus criteria for lifelong BD (DSM-IV, ICD-9, or ICD-10). The GWAS meta-analysis identified 64 independent genetic loci associated with BD, 33 of which were newly discovered, demonstrating genome-wide significance (*P* < 5 × 10^−8^).

### 2.3 Immunity-wide GWAS data sources

The GWAS summary statistics for each immunophenotype can be obtained from the GWAS catalog, with accession numbers ranging from GCST90001391 to GCST90002121, including a cohort of 3,757 Sardinians (34). A high-density array, based on a reference panel of Sardinian sequences (35), was used to estimate approximately 22 million SNPs and tested for correlation after adjusting for covariates such as age, age2, and sex. A total of 731 immunophenotypes were examined, comprising relative cell counts (192), morphologic parameters (32), absolute cell counts (118), and median fluorescence intensities representing surface antigen levels (389).

### 2.4 Selection of instrumental variables

Based on recent studies, we set the threshold for SNPs related to immune cell phenotypes at *P* < 1 × 10^-5^ (34, 36). For SNPs related to BD, we used a threshold of *P* < 5 × 10^-8^ (33, 37). Additionally, we conducted a Linkage Disequilibrium check on the aforementioned SNPs (r² = 0.001 and kb = 10,000) (38). Each IV was assessed based on its F-statistic, and only those with F-statistics exceeding 10 were retained for subsequent analysis to ensure robustness and minimize bias from weak instrumental variables (39). SNP harmonization was conducted between the exposure and outcome datasets to maintain consistency in effect allele estimation. Furthermore, SNPs potentially confounded by other variables were filtered out utilizing the Phenoscanner V2 website (http://www.phenoscanner.medschl.cam.ac.uk/).

### 2.5 Statistical analysis

To investigate the causal association between immune cell phenotypes and the risk of bipolar disorder, this study predominantly used the TwoSampleMR package in R (version 4.3.2). The main MR analysis methods were MR Egger, Weighted Median, Inverse Variance Weighted (IVW), Simple Mode, Weighted Mode, and MR-Presso. Among these, the IVW method served as the principal causal effect estimator in MR research due to its robust capability of detecting causality and yielding substantial testing efficacy (40).

In this study, we used a combination of MR-Egger and MR-Presso to assess horizontal pleiotropy (*P* < 0.05 indicating horizontal pleiotropy). Heterogeneity of IVs was measured using Cochran’s IVW Q statistics (*P* < 0.05 indicating heterogeneity). Furthermore, scatter plots and funnel plots were employed. Moreover, leave-one-out sensitivity analyses were conducted to determine if a single SNP influenced the observed causal relationship. Additionally, FDR correction was performed using the Bioladder web tool, as multiplex testing increases the likelihood of type 1 errors (41). To investigate reverse causation, identical techniques were applied to analyze the reverse MR of immune cell morphologies and bipolar disease. Results were considered highly significant when *P* < 0.01 (42). Additionally, based on prior research, an FDR < 0.2 was considered indicative of a causal relationship (43).

## 3 Results

### 3.1 Exploration of the causal effect of immunophenotypes on BD

**Figure 2:**
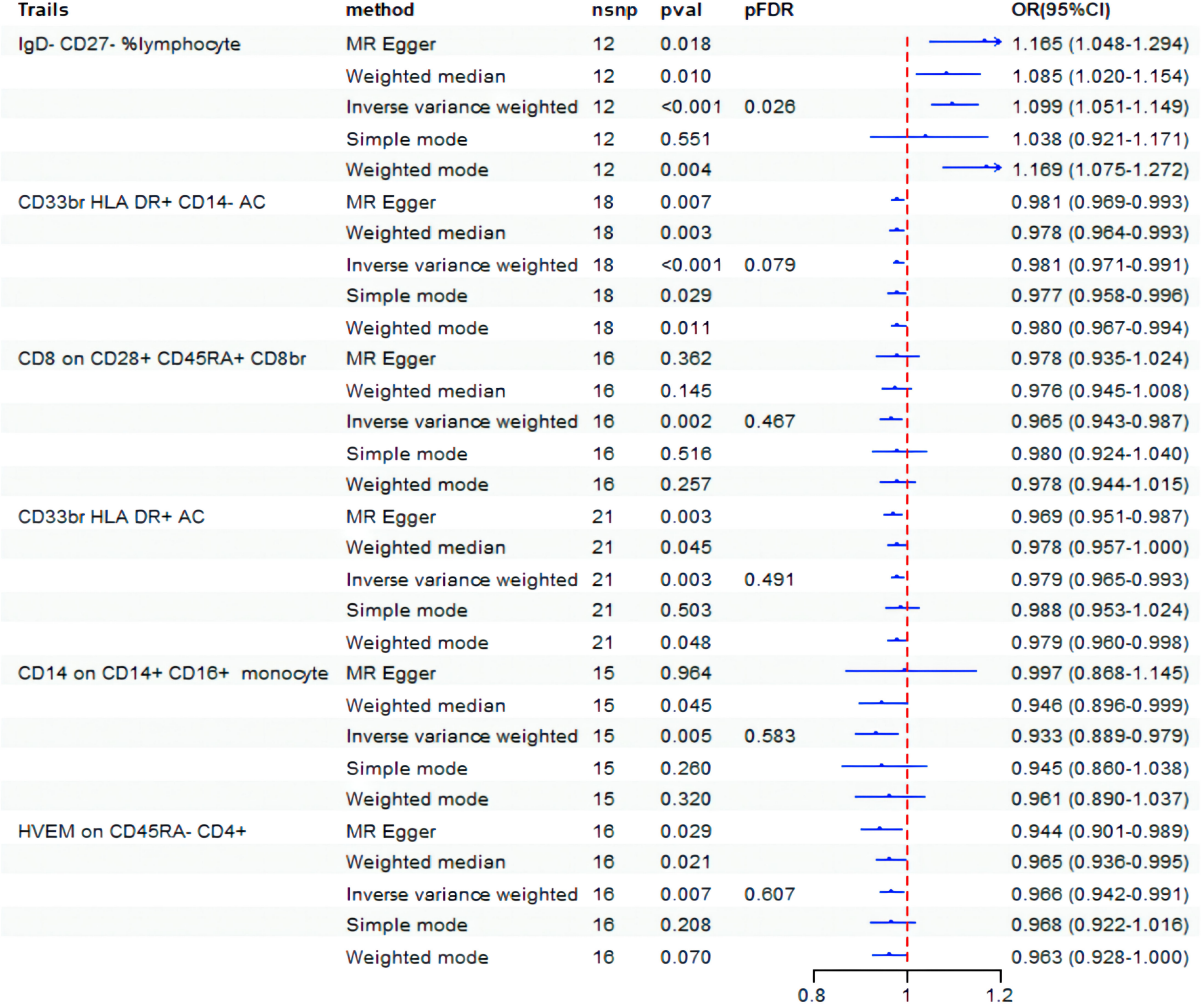
Forest plot showed the causal association between immune cell traits and BD. nsnp, nonsynonymous single-nucleotide polymorphism; OR, odds ratio; CI, confidence interval; PFDR, P value corrected by FDR.

Initially, the causative impact of 731 immunophenotypes as exposure variables on BD was investigated, with findings displayed in **Figure 2**. Before FDR correction, six immunophenotypes were found to impact the occurrence of BD (*P*< 0.01). Specifically, significant negative associations with BD risk were observed for CD33br HLA DR+ CD14-AC on monocyte cells (OR=0.981, 95% CI: 0.971-0.991, *P* = 2.17E-04), CD8 on CD28+ CD45RA+ CD8br on Treg cells (OR=0.965, 95% CI: 0.943-0.987, *P* = 0.002), CD33br HLA DR+ AC on monocyte cells (OR=0.979, 95% CI: 0.965-0.993, *P* = 0.003), CD14 on CD14+ CD16+ monocyte (OR=0.933, 95% CI: 0.889-0.979, *P* = 0.005), and HVEM on CD45RA-CD4+ on maturation stages of T cells (OR=0.966, 95% CI: 0.942-0.991, *P* = 0.007), while IgD-CD27-% lymphocyte on B cells (OR=1.099, 95% CI: 1.051-1.149, *P* = 3.51E-05) showed a significant positive correlation. After adjusting the FDR to 0.2, the risk of BD remained significantly associated with IgD-CD27-% lymphocyte (*P* = 3.51E-05, FDR = 0.026) and CD33br HLA DR+ CD14-AC (*P* = 2.17E-04, FDR = 0.079).

### 3.2 Exploration of the causal effect of BD on immunophenotypes

**Figure 3:**
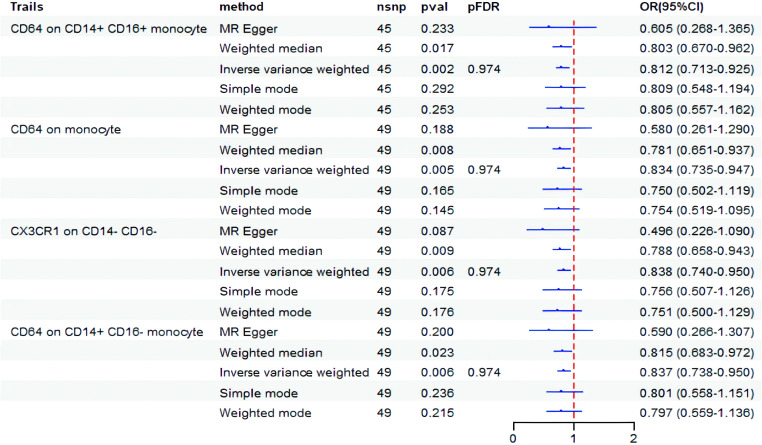
Forest plot showed the causal association between BD and immune cell traits. nsnp, nonsynonymous single-nucleotide polymorphism; OR, odds ratio; CI, confidence interval; PFDR, P value corrected by FDR.

Next, we conducted an analysis to examine the causal impact of BD as an exposure variable on 731 immunophenotypes. The results indicated that BD influences four specific immunophenotypes of monocytes, as shown in **Figure 3**. BD exhibited a negative correlation with CD64 on CD14+ CD16+ monocyte (OR = 0.812, 95% CI: 0.713-0.925, *P* = 0.001), CD64 on monocyte (OR=0.834, 95% CI: 0.735-0.947, *P* = 0.005), CX3CR1 on CD14- CD16- (OR = 0.838, 95% CI: 0.740-0.950, *P* = 0.006), and CD64 on CD14+ CD16- monocyte (OR = 0.837, 95% CI: 0.738-0.950, *P* = 0.006). However, after FDR correction, no statistically significant results were observed.

### 3.3 Sensitivity analysis

**Figure 4:**
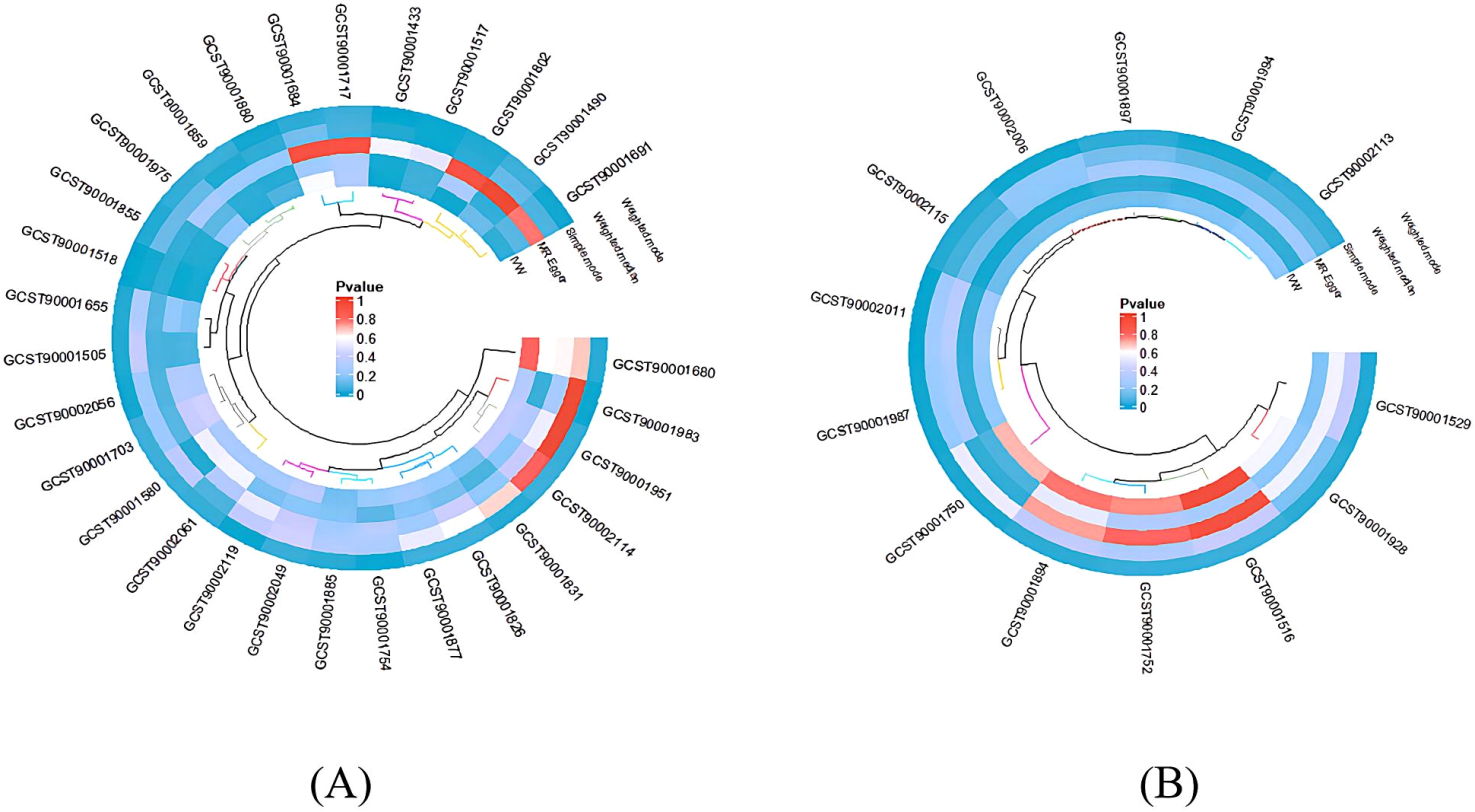
The heatmap depicting the IDs of immune cell phenotypes with positive results and the p-values from the sensitivity analysis. The outer circle represents the IDs of immune cell phenotypes, while the inner circle uses different colors to indicate the p-values of different sensitivity analysis results. **(A)** IDs showing the causal effect of immune cells on BD; **(B)** IDs showing the causal effect of BD on immune cells.

In sections 3.1 and 3.2, the MR-Egger and MR-Presso methods indicated no evidence of horizontal pleiotropy, and the Cochran Q test showed no heterogeneity. These findings are presented in **Supplementary Tables 1**, **2**. Subsequently, scatter plots (**Supplementary Figures 1** and **2**) and funnel plots (**Supplementary Figures 3** and **4**) further substantiated these findings. Furthermore, the leave-one-out analysis demonstrated the robustness of the MR results (**Supplementary Figures 5** and **6**), as excluding any single SNP associated with immunophenotypes and BD did not significantly alter the overall findings. Additionally, all IDs with positive outcomes for immune cell morphologies were filtered out using the IVW technique (*P* < 0.05). A heatmap was used to visually analyze the study findings (**Figure 4**).

## 4 Discussion

Through a two-sample bidirectional MR study, we identified six immune cell phenotypes that exhibited a significant association with the risk of BD (*P*< 0.01). Upon adjusting for FDR < 0.20, two of these immune cell morphologies remained associated with the risk of BD. In the reverse MR analysis, our findings suggested a potential causal relationship between BD and four monocyte immune cell phenotypes (*P*< 0.01). However, after applying FDR correction, none of these associations retained statistical significance.

The current research indicates that B-cell activating factor and A proliferation-inducing ligand, which are critical growth factors for B cells and B cell-driven autoimmunity, exhibit aberrant plasma levels in BD patients. This suggests a pivotal role for B cells in BD (44). Neuroinflammation is one of the causes of BD (45), with B cells facilitating inflammatory responses by expressing inflammation-related genes and ribosomal protein genes (46). B cells can be categorized into four principal subsets based on the differential expression of the immunoglobulins IgD and CD27. IgD−CD27− B cells constitute a heterogeneous population of B cells, which have been shown to be associated with aging and systemic lupus erythematosus (47). Our findings suggest that IgD-CD27-% lymphocytes in B cells might confer a protective effect in BD patients. The CD27-IgD-B cell subset exhibited pronounced pro-inflammatory effects in nonagenarian cells, which were competent to produce pro-inflammatory cytokines (such as TNF, IL-6, and IL-8) (48). However, an analysis of immunosenescence markers and clinical characteristics in BD reveals a tenuous correlation between the percentage of late differentiated B cells (CD3‐CD19+IgD‐CD27‐) and immunological age factors (49). Consequently, the connection between CD27-IgD-cell subsets and BD warrants further exploration.

Treg cells are essential for modulating peripheral immune responses, monitoring the brain’s immune system, and facilitating neuroimmune interactions and coordination (50). Dysfunctional Tregs can lead to abnormal immune activation and neuroinflammation. Increased Treg levels are associated with symptom improvement in mental disorders, while decreased levels correlate with symptom worsening and heightened neuroinflammation (51). In BD patients, TLR-2 and other TLR signaling pathways impair Treg functionality, contributing to neuroinflammation and immune dysregulation (52). Meta-analysis evidence supports that heightened IL-10 levels are associated with increased Treg cells activity in BD (53). Conversely, literature reviews suggest that reductions in Tregs and diminished IL-10 secretion in BD may account for the elevated incidence of autoimmune disorders (54). These observations underscore the potential impact of Treg cells on BD. Our research delineates a negative association between CD8 on CD28+ CD45RA+ CD8br of Treg cells and BD. Emerging studies reveal that during different stages of BD, there is a marked decline in the functional capacities of both Treg cells and effector T cells. In individuals with BD, there is a pronounced reduction in Treg cells expressing CD152 and GARP, along with decreased numbers of CD4+ and CD8+ cells marked by CD71+ (55). CD4+ T cells secrete cytokines to facilitate immune responses, while CD8+ T cells execute cytotoxic activities. Their dysregulation may play a significant role in the pathophysiology of BD (21). Research further indicates that BD patients affected by childhood maltreatment exhibit elevated CD8+ T cells levels compared to their non-maltreated counterparts (56). Additionally, studies have shown correlations between modifications in brain structure and changes in CD4+ and CD8+ cells in individuals with BD (19). Moreover, research suggests that the latent state of HCMV may intensify the immune risk phenotypes associated with BD (55). This aligns with our findings that certain maturation stages of T cells, particularly HVEM expression on CD45RA-CD4+, constitute risk factors for BD.

The research identified CD33br HLA DR+ AC and CD33br HLA DR+ CD14-AC in myeloid cells as risk factors for BD. Previous studies have indicated that common genetic variations at the HLA loci are implicated in increasing susceptibility to BD (57). Moreover, gene expression patterns related to immature neutrophils, a component of myeloid cells, have been observed in peripheral blood cells from patients diagnosed with schizophrenia and bipolar disorder, indicating alterations in the innate immune system (58). Investigations into brain tissue have corroborated some genetic findings associated with BD, such as reduced expression of HLA-DPA1 (59), which encodes the α-chain, while HLA-DR encodes the β-chain, and these chains form the HLA class II molecule. Empirical evidence suggests that the percentages of CD11b/CD33+ hi and CD11b/CD33+ lo cells differed significantly between T carriers and healthy controls in patients with bipolar I disorder in the Han Chinese population (22). This suggests, for the first time, that myeloid-derived suppressor cells might play a role in patients with bipolar I disorder in the Han Chinese population. Thus, further exploration into the association between HLA-DR on myeloid cells and BD is warranted.

Additionally, this study reveals that CD14 expression on CD14+ CD16+ monocytes may impact BD patients, while BD may influence four phenotypic characteristics of monocytes: CD64 on CD14+ CD16- monocytes, CX3CR1 on CD14- CD16- monocytes, CD64 on monocytes, and CD64 on CD14+ CD16+ monocytes. A study using flow cytometry detected differential protein signaling in monocytes between treatment responders and non-responders (60). Pediatric BD patients displayed significantly higher monocyte counts compared to controls, suggesting potential clinical benefits of early BD detection through monocyte counts (61). Monocytes are classified into three categories based on surface markers CD14 and CD16: classical (CD14+CD16-), non-classical (CD14-CD16+), and intermediate (CD14+CD16+) (34). Monocytes exacerbate inflammation by releasing pro-inflammatory cytokines such as IL-10 and IL-15, and this inflammatory activation is closely linked to BD (62). It has been found that BD patients exhibit alterations in the expression of the monocyte marker CD14, and lithium treatment has been noted to exert an immunomodulatory effect on CD14 monocytes and dendritic cells in these patients (20). However, a study found no changes in CD64 mRNA expression among BD patients (63), presenting a possible contradiction to these observations.

In conclusion, the MR analysis has elucidated immune cell phenotypes associated with BD, highlighting immune system activation, inflammation, and bidirectional communication between the brain and the immune system. Neuroinflammation represents one of the key biological mechanisms in BD (64). Immune dysfunction may activate the hypothalamic-pituitary-adrenal axis through various pro-inflammatory cytokines and chemokines, thereby altering cerebral pathways associated with serotonin and catecholamines, leading to mood alterations (65). Furthermore, immune dysregulation may advance the progression of BD by impacting neurotransmitter synthesis, synaptic functionality, neural plasticity (61), brain regions, and neural circuits (66). Additionally, immune dysfunction might disrupt central nervous system operations by modifying the permeability of the blood-brain barrier (67). In turn, the brain can regulate immune responses by controlling the production and release of inflammatory mediators (such as IL-6 and TNF), activating microglia that influence oxidative stress, and modulating the autonomic nervous system (68, 69). Recent studies indicate that epigenetic alterations, including DNA methylation, histone modification, and non-coding RNAs (e.g., miRNA and lncRNA), can induce gene expression variability (70–72). These modifications can be triggered by environmental factors like lifestyle and traumatic experiences, consequently activating pertinent genes and fostering the development of BD (72). Therefore, further investigation into the interplay between inflammation, the immune system, and the brain against a genetic backdrop is crucial for a deeper understanding of the pathophysiological mechanisms of BD and identifying novel therapeutic targets.

However, this research has several limitations. Firstly, due to constraints in the dataset, certain immune cell morphologies could not be analyzed. Secondly, this cohort study, based on a population from Sardinia, lacks stratification by gender and age, limiting the generalizability and accuracy of the conclusions. Finally, the selection of instrumental variables for immune cell phenotypes (*P* < 1×10^−5^) and the interpretation of the results (FDR < 0.2) lacked stringent rigor. Therefore, future research should incorporate larger sample sizes and employ more comprehensive causal analysis techniques (such as CAUSE) to further elucidate the relationship between immune cell phenotypes and BD.

## 5 Conclusion

This study reveals a causal relationship between immune cell phenotypes and bipolar disorder, providing new insights into the pathogenic mechanisms of BD and potential therapeutic targets.

## Data Availability

The original contributions presented in the study are included in the article/**Supplementary Material**. Further inquiries can be directed to the corresponding author.
