# Supplementary material for: Causal role of immune cells in bipolar disorder: a Mendelian randomization study
Source: Front Psychiatry. 2024 Aug 16;15:1411280. doi: 10.3389/fpsyt.2024.1411280 (PMC11362081; doi:10.3389/fpsyt.2024.1411280)
Supplement: Supplementary file 1 [file Image1.pdf]

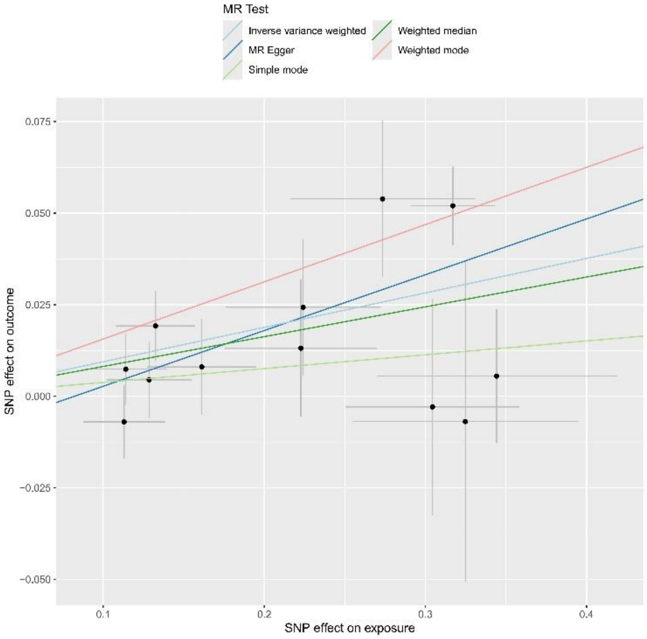
MR Test
Inverse variance weighted
MR Egger
Simple mode
Weighted median
Weighted mode
0.075
0.050
0.025
0.000
−0.025
−0.050
0.1
0.2
0.3
0.4
SNP effect on outcome
SNP effect on exposure

(A)

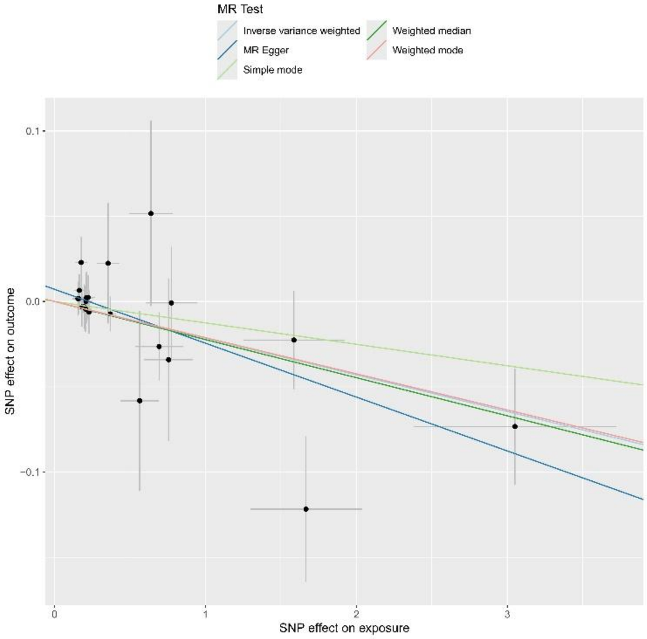
MR Test
Inverse variance weighted
MR Egger
Simple mode
Weighted median
Weighted mode
0.1
0.0
−0.1
0
1
2
3
SNP effect on outcome
SNP effect on exposure

(B)

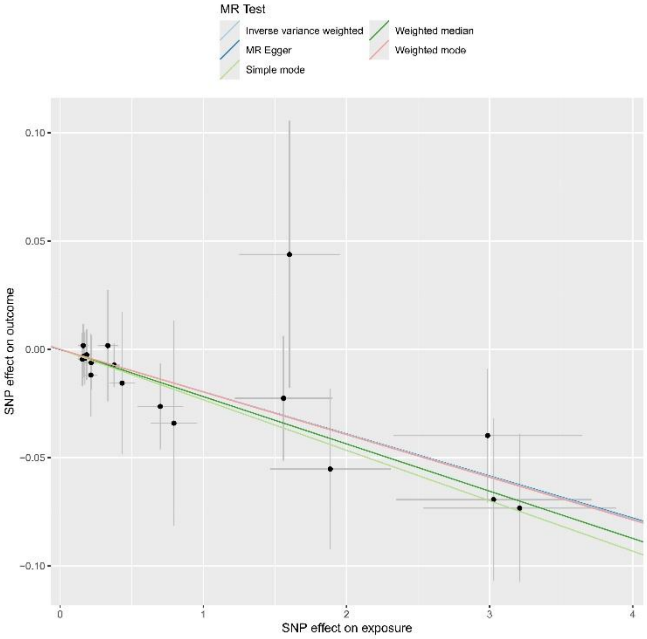
MR Test
Inverse variance weighted
MR Egger
Simple mode
Weighted median
Weighted mode
0.10
0.05
0.00
-0.05
-0.10
0
1
2
3
4
SNP effect on outcome
SNP effect on exposure

(C)

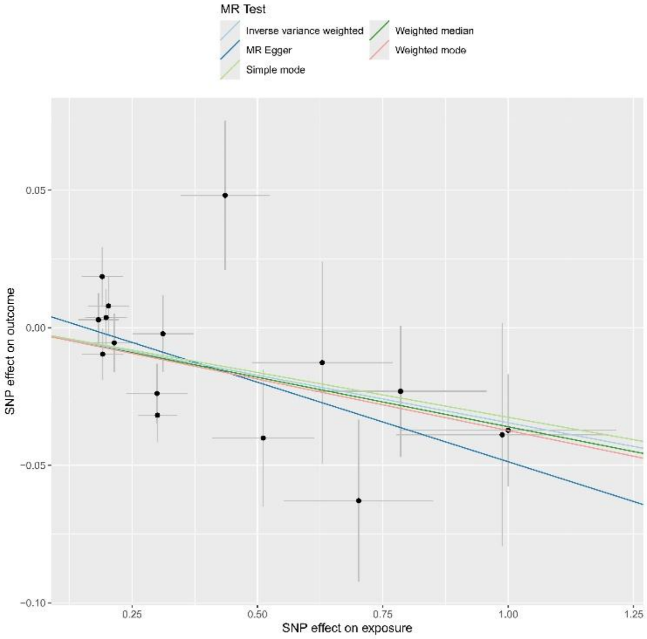
MR Test
Inverse variance weighted
MR Egger
Simple mode
Weighted median
Weighted mode
0.05
0.00
−0.05
−0.10
0.25
0.50
0.75
1.00
1.25
SNP effect on outcome
SNP effect on exposure

(D)

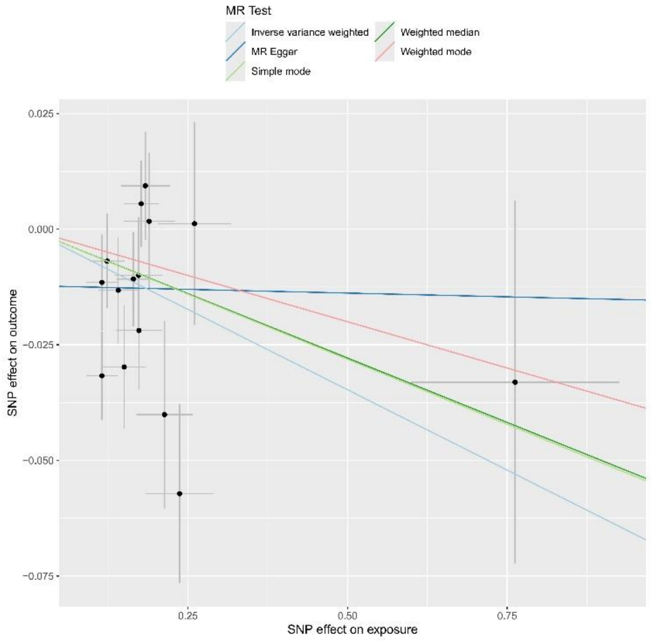

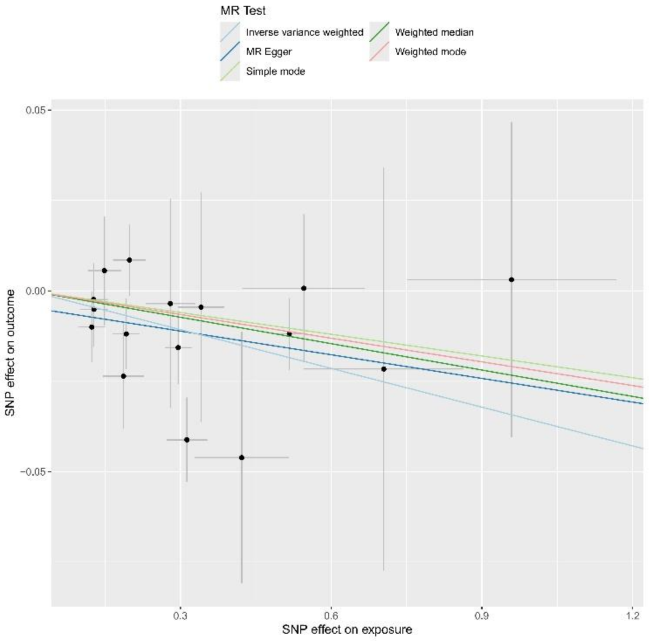

(E) **(F)**

Supplementary Figure 1. Scatter plots of the causal effects of immune cells on BD.**(A).** Scatter plot between IgD- CD27- %lymphocyte and BD risk;**(B).** Scatter plot between CD33br HLA DR+ AC and BD risk; **(C).** Scatter plot between CD33br HLA DR+ CD14- AC and BD risk; **(D).** Scatter plot between HVEM on CD45RA- CD4+ and BD risk; **(E).** Scatter plot between CD14 on CD14+ CD16+ monocyte and BD risk; **(F).** Scatter plot between CD8 on CD28+ CD45RA+ CD8br and BD risk.

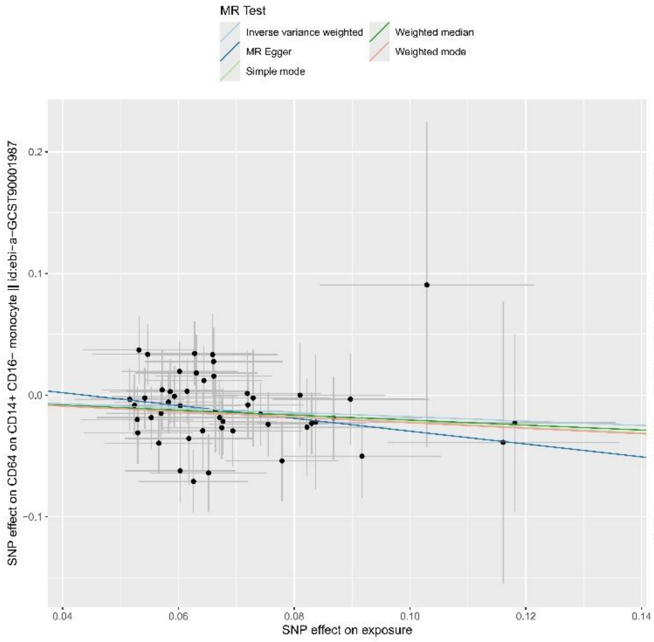

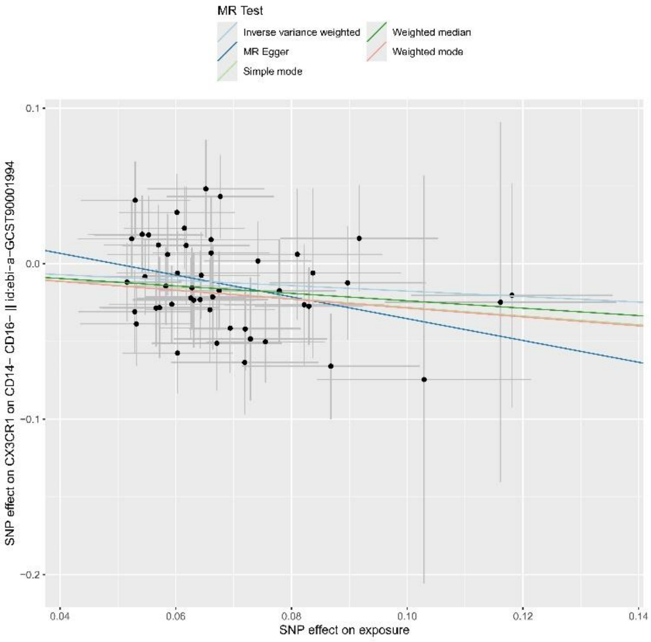

(A) **(B)**

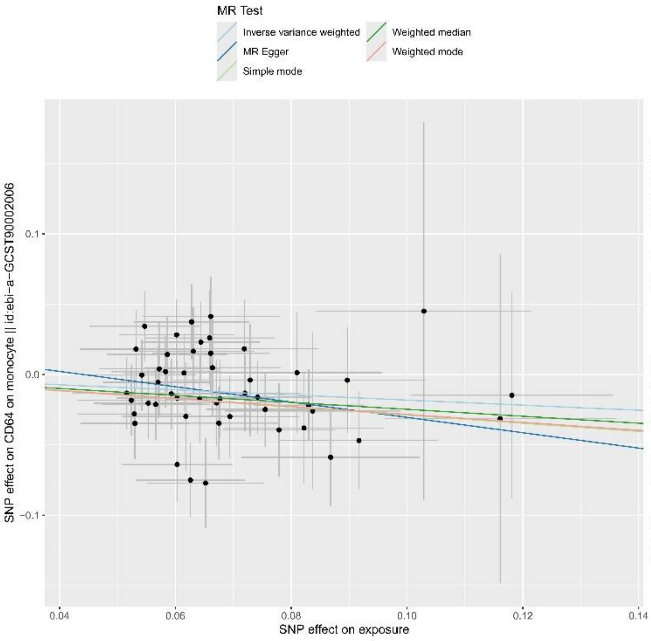

(C)

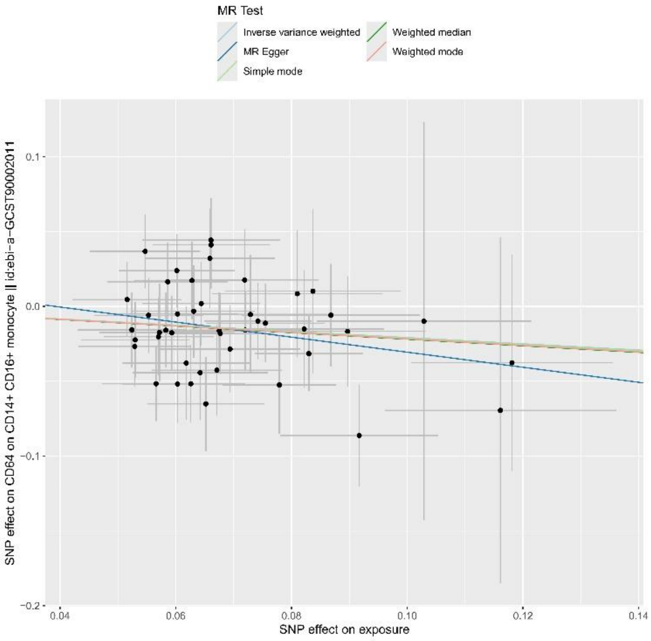

(D)

Supplementary Figure 2. Scatter plots of the causal effects of BD on immune cells. **(A).** Scatter plot between BD risk and CD64 on CD14+ CD16- monocyte;**(B).** Scatter plot between BD risk and CX3CR1 on CD14- CD16-; **(C).** Scatter plot between BD risk and CD64 on monocyte; **(D).** Scatter plot between BD risk and CD64 on CD14+ CD16+ monocyte.

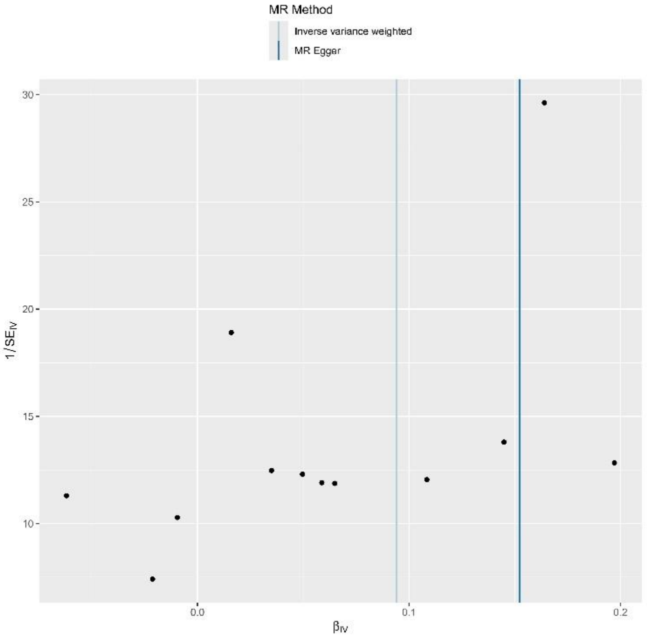

(A)

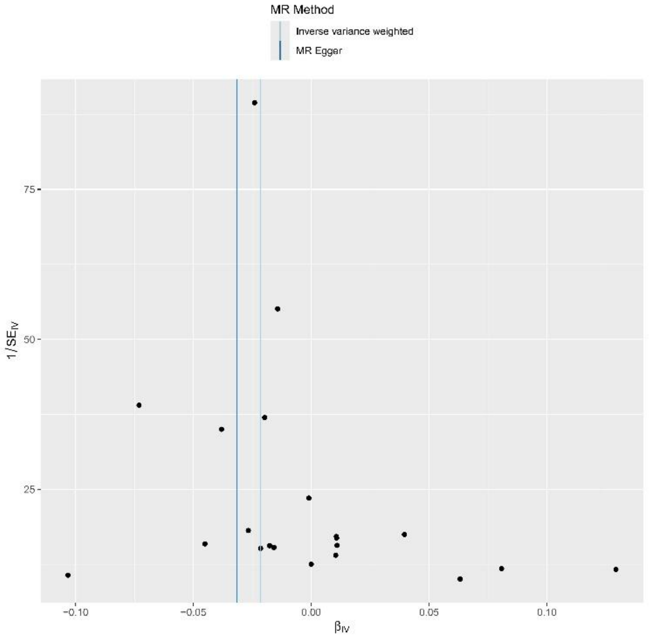

(B)

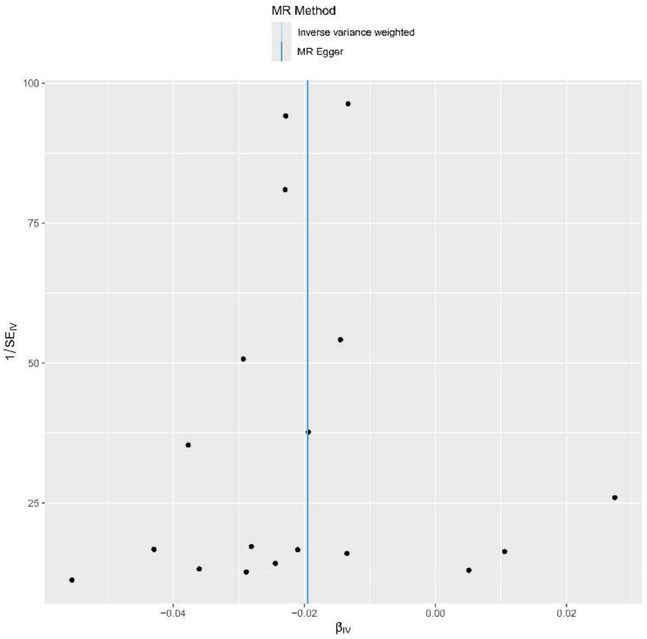

(C)

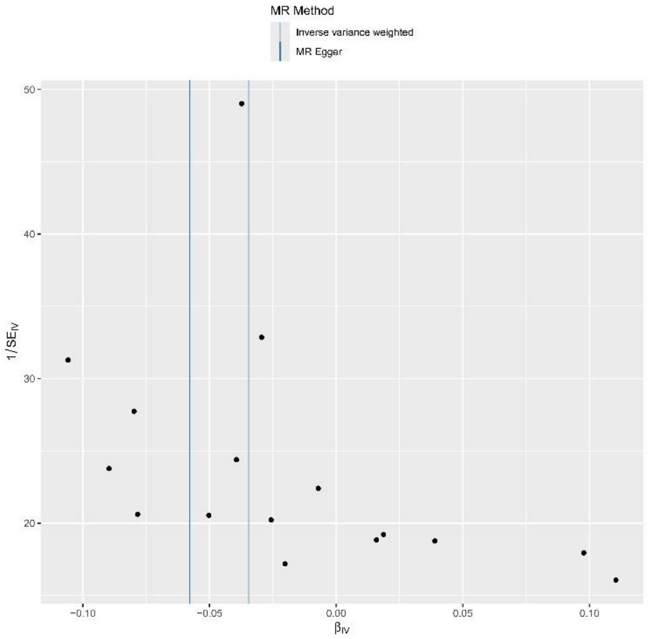

(D)

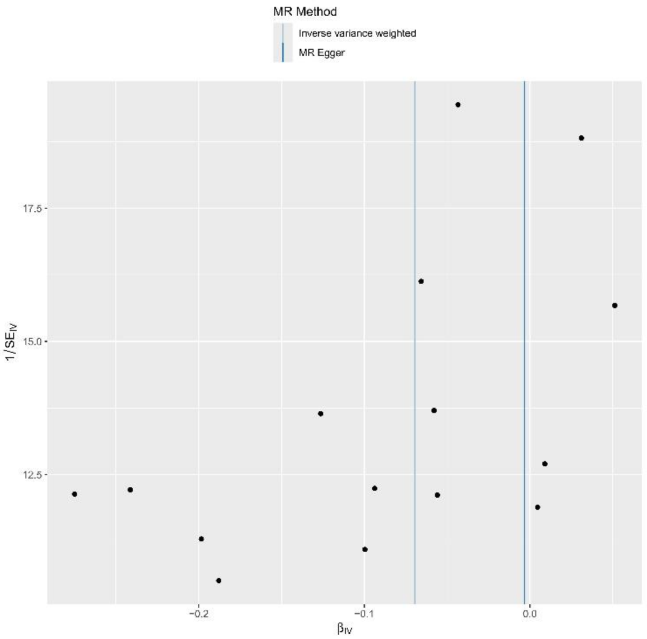

(E)

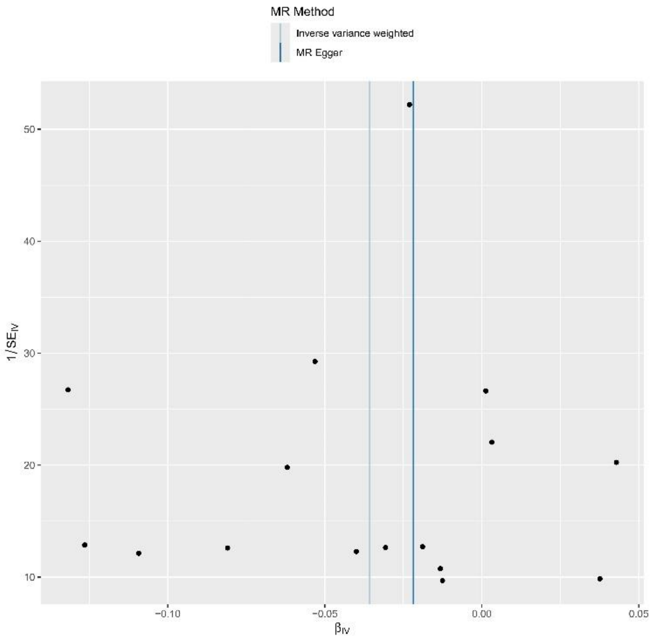

(F)

Supplementary Figure 3. Funnel plots of the causal effects of immune cells on BD.**(A).** Funnel plot between IgD- CD27- %lymphocyte and BD risk;**(B).** Funnel plot between CD33br HLA DR+ AC and BD risk; **(C).** Funnel plot between CD33br HLA DR+ CD14- AC and BD risk; **(D).** Funnel plot between HVEM on CD45RA- CD4+ and BD risk; **(E).** Funnel plot between CD14 on CD14+ CD16+ monocyte and BD risk; **(F).** Funnel plot between CD8 on CD28+ CD45RA+ CD8br and BD risk.

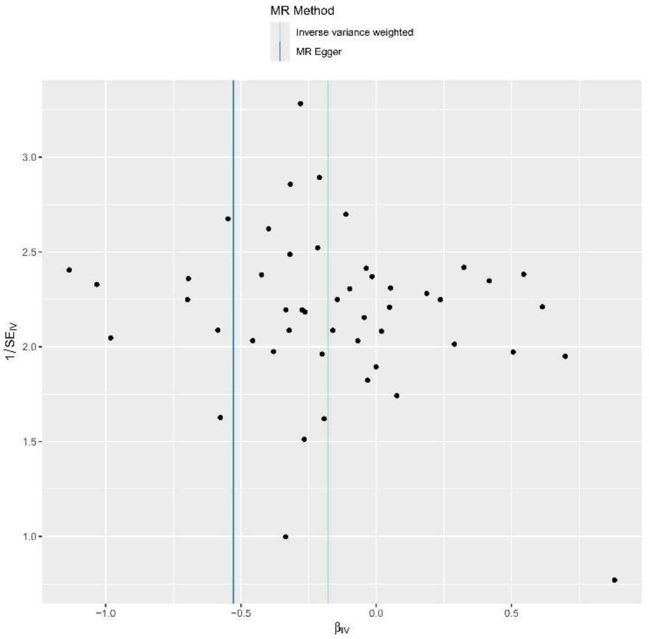

(A)

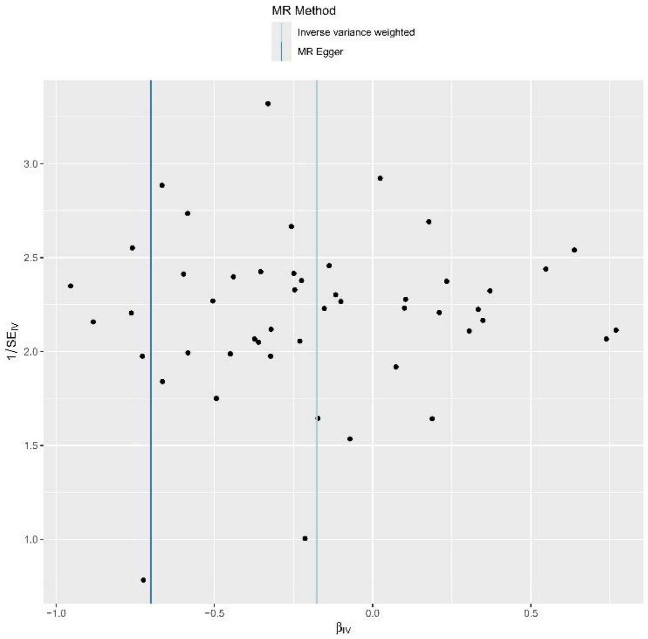

(B)

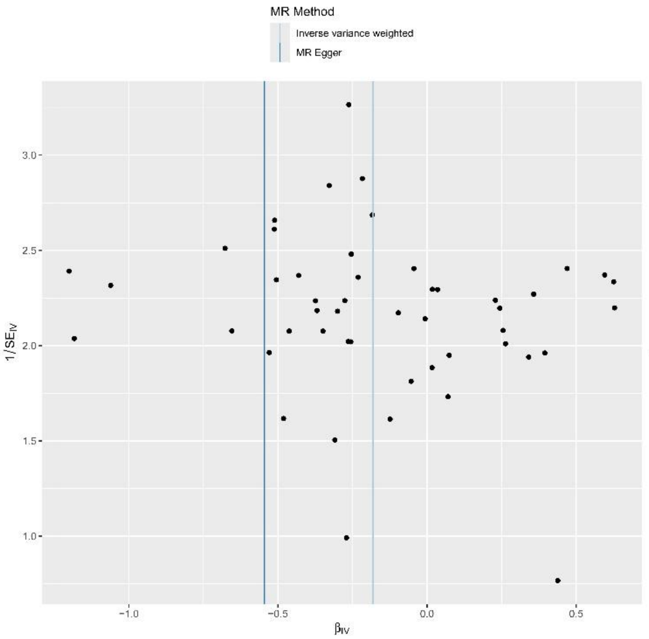

(C)

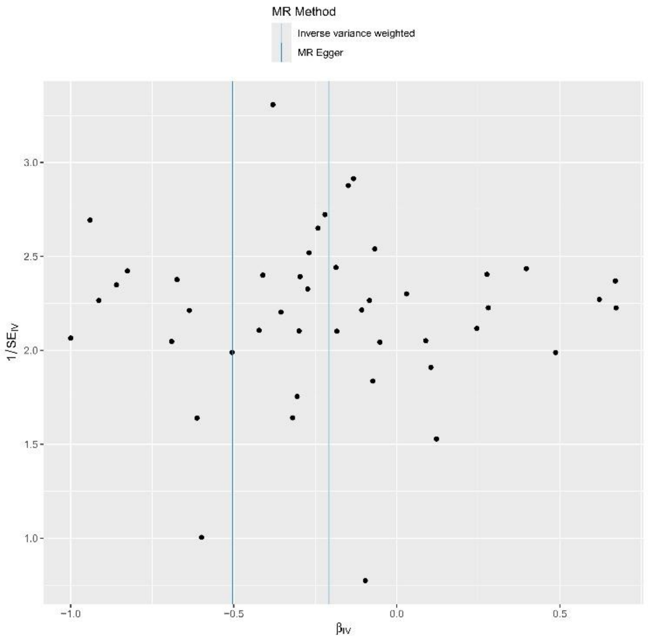

(D)

Supplementary Figure 4. Funnel plots of the causal effects of BD on immune cells. **(A).** Funnel plot between BD risk and CD64 on CD14+ CD16- monocyte;**(B).** Funnel plot between BD risk and CX3CR1 on CD14- CD16-; **(C).** Funnel plot between BD risk and CD64 on monocyte; **(D).** Funnel plot between BD risk and CD64 on CD14+ CD16+ monocyte.

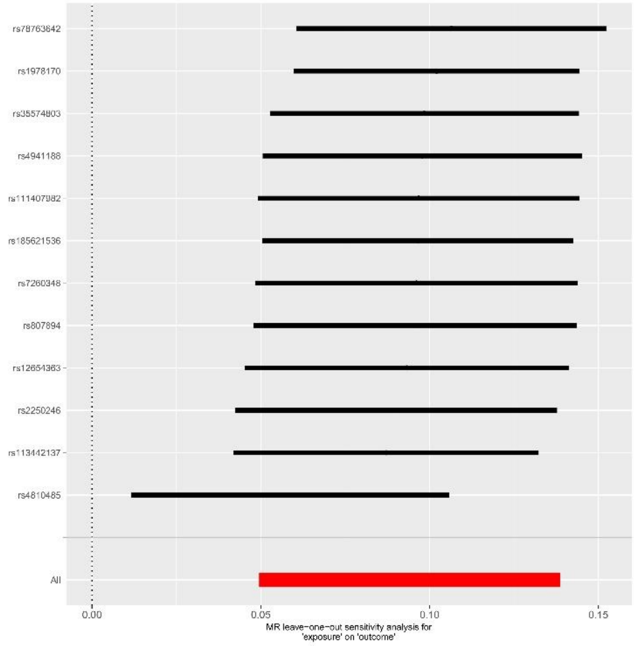
rs78763642
rs1978170
rs35574803
rs4941188
rs111407982
rs185621536
rs7260348
rs807894
rs12654363
rs2250246
rs113442137
rs4810485
All
0.00
0.05
0.10
0.15
MR leave-one-out sensitivity analysis for
'exposure' on 'outcome'

(A)

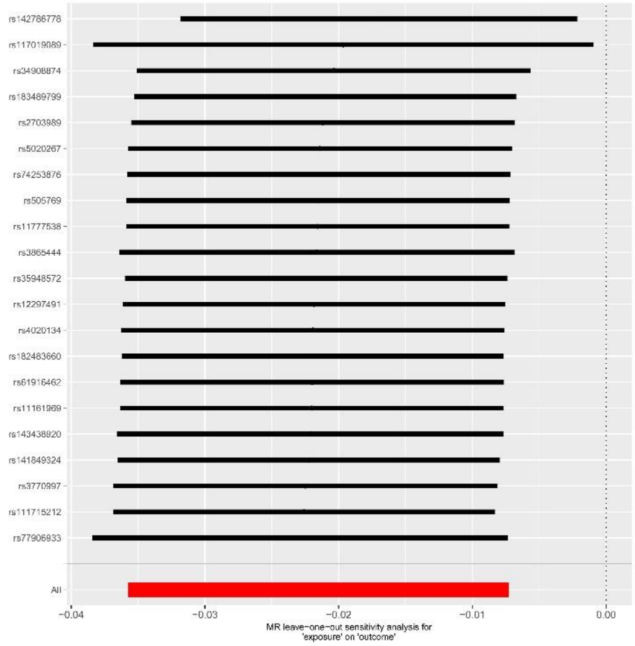
rs142786778
rs117019089
rs34908874
rs183489799
rs2703989
rs5020267
rs74253876
rs505769
rs11777538
rs3865444
rs35948572
rs12297491
rs4020134
rs182483660
rs61916462
rs11161969
rs143438920
rs141849324
rs3770997
rs111715212
rs77906933
All
−0.04
−0.03
−0.02
−0.01
0.00
MR leave-one-out sensitivity analysis for
'exposure' on 'outcome'

(B)

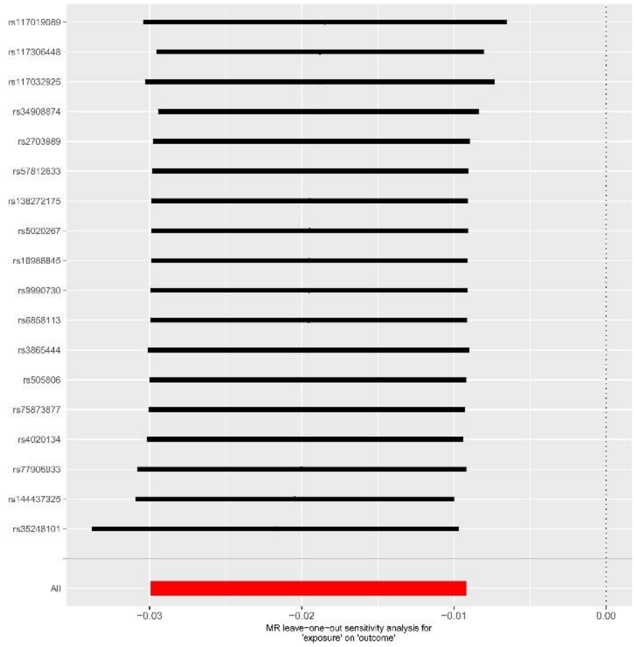
rs117019089
rs117306448
rs117032925
rs34908874
rs2703989
rs57812633
rs138272175
rs5020267
rs16988845
rs9990730
rs6858113
rs3865444
rs505806
rs75873877
rs4020134
rs77906933
rs144437325
rs35248101
All
−0.03
−0.02
−0.01
0.00
MR leave-one-out sensitivity analysis for
'exposure' on 'outcome'

(C)

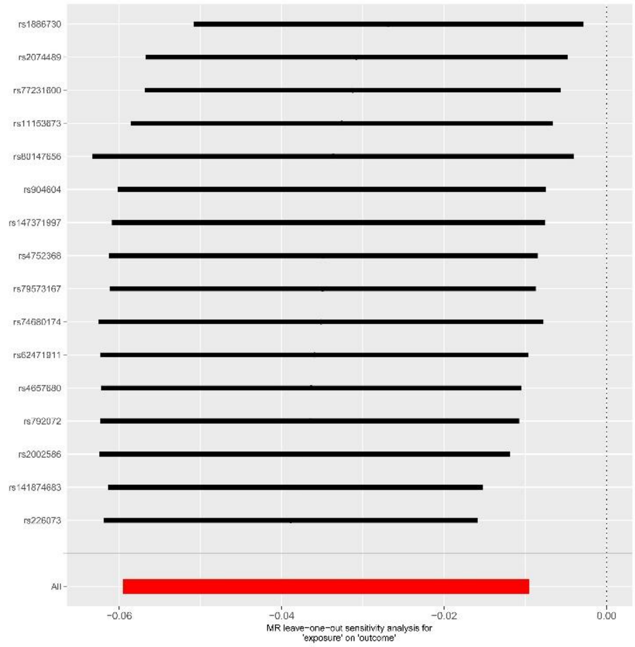
rs1886730
rs2074489
rs77231600
rs11153873
rs80147656
rs904604
rs147371997
rs4752368
rs79573167
rs74680174
rs62471911
rs4657680
rs792072
rs2002586
rs141874683
rs226073
All
−0.06
−0.04
−0.02
0.00
MR leave-one-out sensitivity analysis for
'exposure' on 'outcome'

(D)

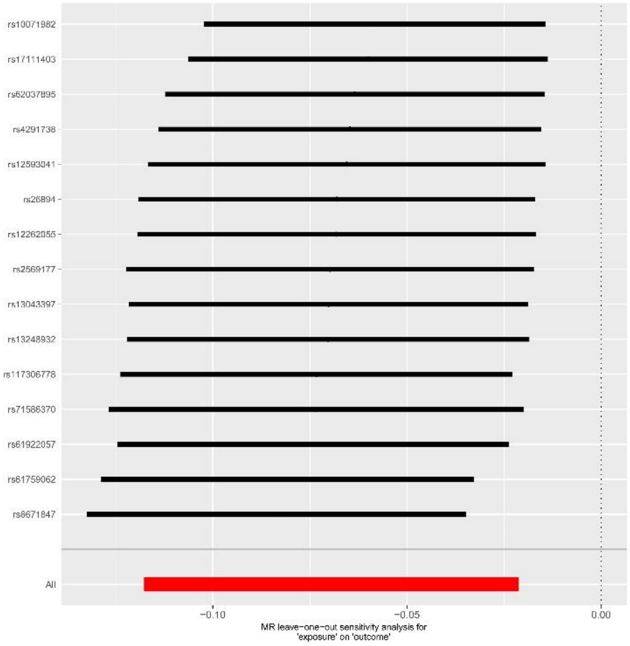

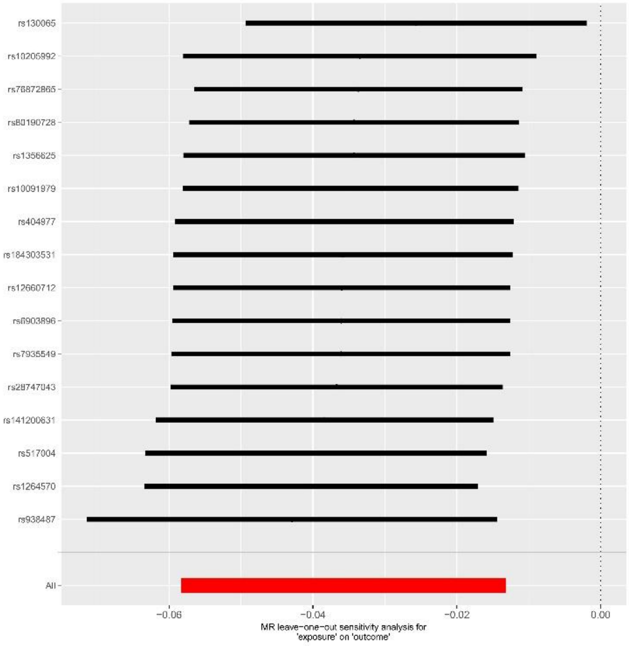

(E) **(F)**

Supplementary Figure 5. Leave-one-out plots of the causal effects of immune cells on BD.**(A).** Leave-one-out plot between IgD- CD27- %lymphocyte and BD risk;**(B).** Leave-one-out plot between CD33br HLA DR+ AC and BD risk; **(C).** Leave-one-out plot between CD33br HLA DR+ CD14- AC and BD risk; **(D).** Leave-one-out plot between HVEM on CD45RA- CD4+ and BD risk; **(E).** Leave-one-out plot between CD14 on CD14+ CD16+ monocyte and BD risk; **(F).** Leave-one-out plot between CD8 on CD28+ CD45RA+ CD8br and BD risk.

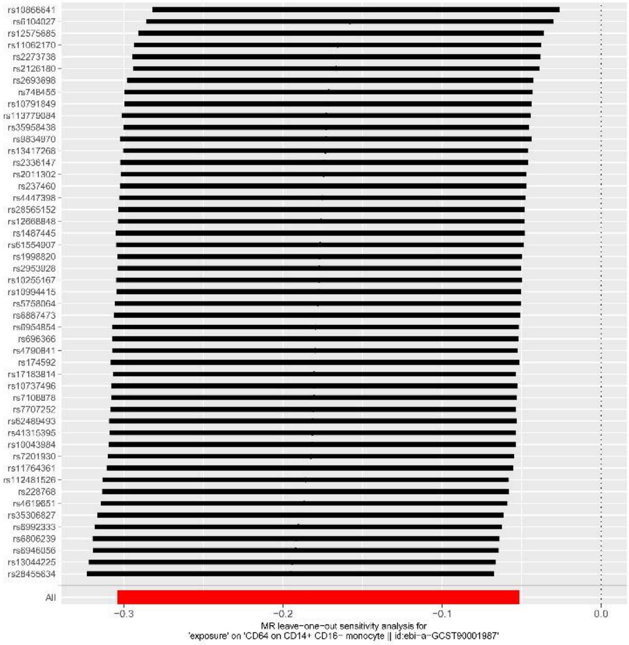

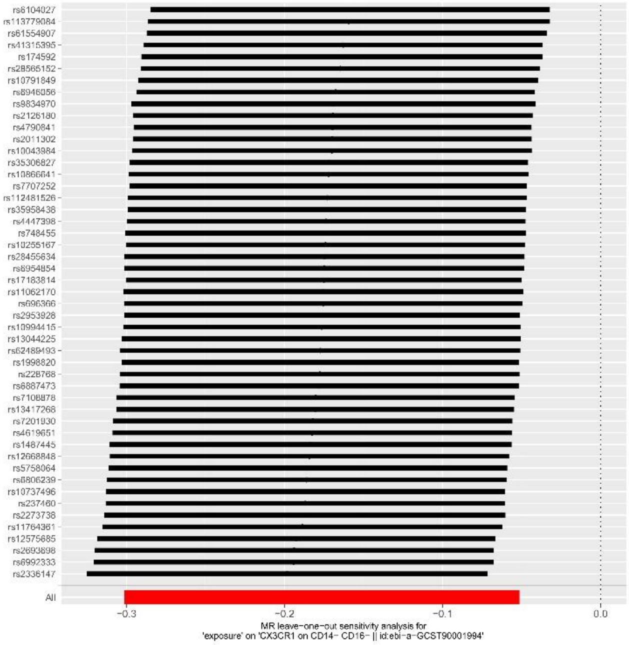

(A) **(B)**

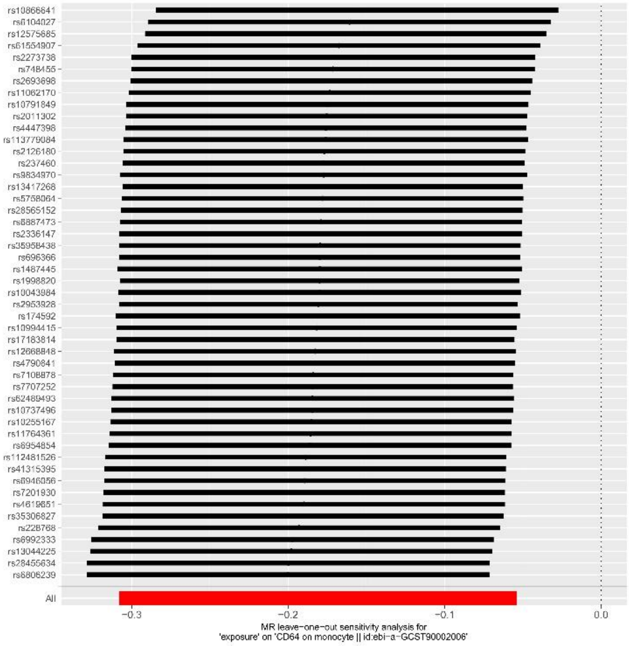

(C)

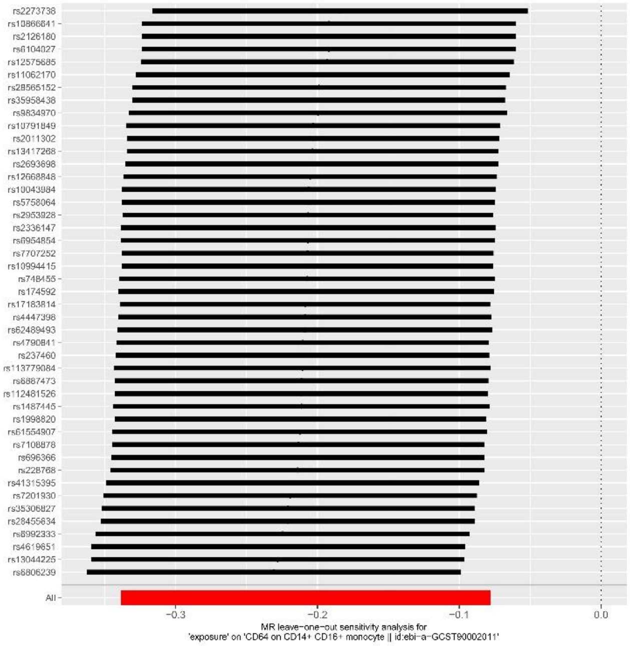

(D)

Supplementary Figure 6. Leave-one-out plots of the causal effects of BD on immune cells. **(A).** Leave-one-out plot between BD risk and CD64 on CD14+ CD16- monocyte;**(B).** Leave-one-out plot between BD risk and CX3CR1 on CD14- CD16-; **(C).** Leave-one-out plot between BD risk and CD64 on monocyte; **(D).** Leave-one-out plot between BD risk and CD64 on CD14+ CD16+ monocyte.
